# Brain proteomics links oxidative stress with metabolic and cellular stress response proteins in behavioural alteration of Alzheimer's disease model rats

**DOI:** 10.3934/Neuroscience.2019.4.299

**Published:** 2019-11-15

**Authors:** Mohammad Azizur Rahman, Shahdat Hossain, Noorlidah Abdullah, Norhaniza Aminudin

**Affiliations:** 1 Department of Biochemistry and Molecular Biology, Jahangirnagar University, Savar, Dhaka, Bangladesh; 2 Mushroom Research Centre, Institute of Biological Sciences, Faculty of Science, University of Malaya, Kuala Lumpur, Malaysia; 3 University of Malaya Centre for Proteomics Research, Faculty of Medicine, University of Malaya, Kuala Lumpur, Malaysia

**Keywords:** Alzheimer's disease, behavioural alteration, oxidative stress, proteomics

## Abstract

Alzheimer's disease (AD) impairs memory and learning related behavioural performances of the affected person. Compared with the controls, memory and learning related behavioural performances of the AD model rats followed by hippocampal proteomics had been observed in the present study. In the eight armed radial maze, altered performance of the AD rats had been observed. Using liquid chromatography coupled tandem mass spectrometry (LC-MS/MS), 822 proteins had been identified with protein threshold at 95.0%, minimum peptide of 2 and peptide threshold at 0.1% FDR. Among them, 329 proteins were differentially expressed with statistical significance (P < 0.05). Among the significantly regulated (P < 0.05) 329 proteins, 289 met the criteria of fold change (LogFC of 1.5) cut off value. Number of proteins linked with AD, oxidative stress (OS) and hypercholesterolemia was 59, 20 and 12, respectively. Number of commonly expressed proteins was 361. The highest amount of proteins differentially expressed in the AD rats were those involved in metabolic processes followed by those linked with OS. Most notable was the perturbed state of the cholesterol metabolizing proteins in the AD group. Current findings suggest that proteins associated with oxidative stress, glucose and cholesterol metabolism and cellular stress response are among the mostly affected proteins in AD subjects. Thus, novel therapeutic approaches targeting these proteins could be strategized to withstand the ever increasing global AD burden.

## 1. Introduction

Alzheimer's disease (AD) is the most common form of dementia, the deteriorated behavioural state that starts with gradual decrement of memory and learning capabilities. This occurs due to degeneration of the neurons associated with memory and learning in the hippocampus. As a consequence, AD patients face problem in remembering, recognizing, positioning and in executing personal activities. At advanced ages, they become solely dependent on their care-givers and family members. AD is posing threat to the global economic policy as it impacts world economy negatively [1]. AD is mainly of two types: familial (mostly inherited, caused by gene mutation, rare in number) and sporadic (occurs mainly at advanced ages, most of the noticed AD cases) [1]. Neuropathological hallmarks of AD involves amyloid plaques and neurofibrillary tangles (NFT) [1]. Amyloid plaques consist mainly of extracellular deposition of amyloid beta (Aβ) peptides [1]. Proteomics of the postmortem amyloid plaques had shown that in addition to Aβ, amyloid plaques harbor about 488 types of proteins [2]. Contrary to the amyloid plaques, NFTs are intra-neuronal aggregates of misfolded and/or hyperphosphorylated tau protein. Proteomics approaches had identified sixty three NFT associated proteins in addition to tau [3]. Besides, neuropil threads and dystrophic neurites containing hyperphosphorylated and misfolded tau proteins, loss of neuronal neuropils and synapses, astrogliosis and microgliosis are other hallmarks of AD. Very often, synaptic and axonal degeneration result in cognitive impairment as well as dendritic atrophy, the retrograde degeneration of axons, and the eventual atrophy of dendrites and perikarya [1].

The biochemical alterations can be examined either in animal models or in AD brain biopsies as the living human brain remains mostly inaccessible towards neurochemical scrutinization [4]. Thus, respite all these biochemical manifestations, study on memory and learning related behavioural performance associated neurochemical manifestations in AD model animals spur high in prognostic, diagnostic and in formulating therapeutic strategy against AD pathogenesis. Both transgenic and non-transgenic AD model animals are *in vogue* worldwide [4]. The transgenic form represents the familial type while the non-transgenic AD model animals manifest the sporadic form of AD [4].

The association between hypercholesterolemia and increased risk of AD pathogenesis had been emanated from multiple studies. Hypercholesterolemia linked increased deposition of Aβ in the rabbit hippocampus was first demonstrated by Sparks et al. (1994) [5]. Later, Refolo et al. (2000) showed that transgenic mice fed hypercholesterolemic diet become much prone to development of AD [5]. In their following studies, Refolo et al. (2000) observed that feeding of cholesterol lowering drugs to the transgenic AD mice reduces the risk of AD development by about 50% [6]. Epidemiological studies suggest 2-3 times greater risk of late-age dementia and AD in people having mid-life hypercholesterolemia than the normo-cholesterolemics [7]. Brain is much prone to oxidative stress (OS) due especially to its high lipid and low anti-oxidative defense arsenal content and its enormous (one fourth of total respired) oxygen utilization [8]. Inside brain, neurons are more vulnerable to OS and direct association between OS and Aβ generation had been observed in both animal models in human subjects [9]. Transgenic AD model mice (Tg19959) having partially defective anti-oxidant enzyme MnSOD demonstrated accelerated OS as well as Aβ level in brain [10]. Two to three-fold overexpression of the same enzyme in the same model animals showed lowered OS (representing 50% increased level of catalase along with 50% reduced level of protein oxidation), about 33% decreased level of Aβ deposition and restoration of memory deficit [11]. In line with these, deficiency of Cu/ZnSOD1 in Tg2576 AD model mice enhanced OS-driven Aβ oligomerization and memory loss [12].

Proteins are highly vulnerable towards OS. OS causes irreversible modification of their secondary and tertiary structures, shape and function. OS induced protein structural modifications include unfolding, subunit dissociation, backbone fragmentation, aggregation and hydrophobic residues exposition [13]. Compared to the normal subjects, the AD brains suffer from higher protein oxidation. Most of the proteins/enzymes oxidative vulnerable in AD brains are those linked with glycolysis and citric acid cycle [14]. As a result, impaired energy production emanates from excessive protein oxidation in the AD brains.

In the present study, a non-transgenic rat model representing the sporadic form of AD prepared by intra-cerebroventricular infusion of Aβ_1-42_ was used followed by memory and learning related behavioural study of the control and AD model rats in eight armed radial maze and hippocampal proteomics analyses of the respective animals [15]. Our hypothesis was that AD model rats would represent cognitive deficiency in behaviour test and altered expression of hippocampal proteins compared with their control counterparts.

## 2. Materials and methods

### 2.1. Animals

Wistar male rats (120 ± 5 gm) were divided into two groups: control (C) and AD (A), each group containing 15 rats. AD model rats were prepared by infusing Aβ_1-42_ (ab120959, abcam, USA) into the cerebral ventricles following the method of Abdullah et al (2013) [15]. Anesthetizing the rats with intra-peritoneal injection of sodium pentobarbital (40 mg/kg body weight), hair on the rats' heads was shaved and fixed the rats in the stereotaxic frame (SR-5R-HT, Narishige, Japan) by using the locks associated with it. Povidone-iodine (6%, USP) was used as antiseptic to rub the shaved portion of the head and placed the head at the midpoint of the stereotaxic frame. The skull was opened through incision, clipped the skin and sterile cotton and ice cold saline (0.9% NaCl) were used to wash bleeding. Removing skull-linked muscles, the skull was kept open and dried for a while so that the bregma became visible. Paralleling the skull to the frame, the ventricular points were stereotaxically spotted at 1.2 mm lateral and 0.8 mm posterior distant from the bregma following the brain atlas of Paxinos and Watson (1998). Marking of the spotted points followed microdrilling to make two pin holes at the two ventricles. For infusion of Aβ_1-42_, a vehicle consisting of 35% acetonitrile (v/v) and 0.1% trifluoroacetic acid (v/v, pH 2.0) was used. Aβ_1-42_ (5 µL, 2.5 nmol at 1 µL/min flow rate) was infused in the right ventricle and 5 µL of 1% Alcl_3_ in the left ventricle (for Aβ_1-42_ aggregation) using a Hamilton microsyringe (Neuros model 7001 KH, Hamilton, USA). After infusion, the rat was left untouched for a while so that adequate absorption could take place and then sealed the pin holes with sterile bone foam and cement. After drying of the bone cement, the skin clips were removed, wetted skin with saline (0.9% saline) and stitched the skin using sterile needle and thread. Thus, the AD positive controls were prepared. A sham control group was prepared using the same solvent injection. In order to be certain about Aβ_1-42_ infusion in the cerebral ventricles, another trial surgery of rats maintaining the same procedure and using coomassie brilliant blue instead of Aβ_1-42_ was performed in parallel with the original. After about 1 hour, rats of the trial group were sacrificed, the brains collected and the ventricles observed. Observing the dye in the right cerebral ventricle following the same procedure, reaching of Aβ_1-42_ to the right cerebral ventricles could be ascertained. Animals had been kept fasting overnight before sacrifice to reduce variability in investigating parameters and to facilitate surgical procedures. All the experimental protocols had been approved by the ethical permission committee, University of Malaya Institutional Animal Care and Use Committee (UMIACUC) [Ethics reference no. ISB/25/04/2013/NA (R)].

### 2.2. Eight-armed radial maze test

#### 2.2.1. Principle

Developed by Olton and Samuelson (1976), the eight armed radial maze had successfully been used in deciphering the spatial learning abilities of the rats [16]. The test is based on the principle of win-shift task: the experimental animal is supposed to achieve the reward (food pellet, as it is kept hungry) from different locations within the environment and it must forage among different goals. While foraging, it uses landmarks and visual cues for memorizing spatial location of the reward and later on utilizes its acquired memory for further navigation. Its repetitive search in the same arm of the maze (working memory error, WME) and failure to achieve the reward in a single trial (reference memory error, RME) are considered as poor or impaired memory.

#### 2.2.2. Eight-armed maze set up

**Figure 1.**
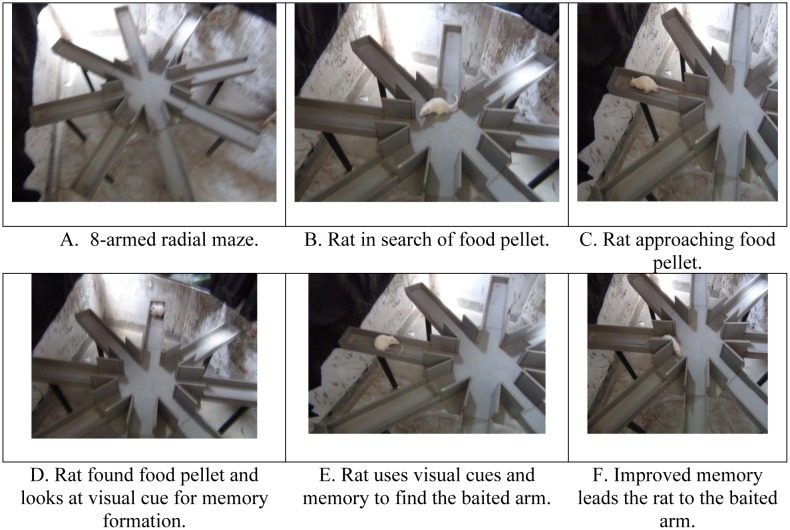
Eight armed radial maze test.

An eight - armed radial maze made of polyvinyl chloride (0.5 cm width) was used in the present experiment (Figure 1). The maze was placed in a closed room and raised 38 inches above from the floor with arms prolonged from a central octagonal arena with a diameter of 30 cm. Each arm of the maze was of 50 cm long, 11 cm wide and 4 cm height. Additional arms (12 cm height) extended from the octagonal arena prevented rats jumping from one arm to another. Two centimeter before the end of each arm, there were food cups of 2.5 cm diameter and 1.5 cm depth (Figure 1). Visual cues (wall posters, chair and tables) and the position of the experimenter behind a canopy were kept constant during the entire period of the experiment.

#### 2.2.3. Behavioural experiment

Behavioural experiments of 8-armed radial maze consisted of three phases: conditioning, acquisition and final experimentation

- Conditioning: Each rat was handled for 5 minutes 7 days to habituate them to the experimenter and to the maze environment. The went up to gradual deprivation of food up to 15% of normal food intake to induce them to search for food as the food pellets (10 mg) were placed in the food cups of the maze.
- Acquisition: Rats placed at the center of the maze explored the arms to find the food reward (Figure 1). They were trained for 7 days with one trial per day. Food pellets placed in any arm was kept constant across the whole session and trial.
- Experimentation phase: Each rat was examined to collect food reward from the food cups of the four of the eight arms (Figure 1). A trial was terminated after either all the bait had been consumed or after 5 minutes had elapsed. Following the memory error definition of Jarrard et al. (1984) [17], we measured three parameters of memory function: RME, WME and maze latency (time required for completing the entry into the four baited arms).

### 2.3. Brain sample preparation and protein quantification

Twenty four hours following the last behavioural test, rats were kept in fasting overnight. Rats were anaesthetized with intra-peritoneal injection of sodium pentobarbital (35 mg/kbw), sacrificed, head removed followed by collection of brain on ice bath. Brains were frozen in liquid nitrogen and stored at −80 °C. Protein extraction from the brain samples was performed following homogenization of the brain sample (50 mg) with lysis buffer (1ml) using a homogenizer (Polytron PT 1200, Kinematica). To avoid protein degradation, we added 10 µL of protease inhibitor cocktail during homogenization followed by centrifugation at 10000× g at 4 °C for 10 minutes. The supernatant was collected and preceded towards delipidation following the methods of Shevchenko et al. (2010) [18]. Later, protein separation through SDS-PAGE and protein quantification through LC-chip MS/MS Q-TOF was performed.

### 2.4. Protein separation through SDS-PAGE

The mini-PROTEAN tetra cell (165-8000, BIO-RAD, USA) was used according to the manufacturer's instructions for running SDS-PAGE in the current study. Coomassie brilliant blue (0.1%) was used for staining the proteins with shaking for about 20 minutes. For destaining, 10% acetic acid solution (aqueous) was used. The gel immersed into the destaining solution contained in a covered box underwent occasional stiring until the entire gel was fully destained. Then, individual bands was cut and if any gel plug still contained stain, repeated shaking of the gel plugs in 50 µL of 50% acetonitrile (ACN) in 50 mM ammonium bicarbonate was continued.

#### 2.4.1. In-gel tryptic digestion

For disrupting the tertiary structures of the solubilized proteins, reduction and alkylation are applied to them so that the disulfide linkages are broken and cannot be re-formed. For reduction, the gel plugs were incubated in 150 µL of 10 mM dithiothreitol (DTT) in 100 mM ammonium bicarbonate buffer at 60 °C for 30 minutes. After cooling at room temperature, the gel plugs were alkylated by incubating in 150 µL of 55 mM iodoacetamide (IAA) in 100 mM ammonium bicarbonate for 20 minutes in the dark chamber. The gel plugs were washed in triplicate with 500 µL of 50% ACN in 100 mM ammonium bicarbonate for 20 minutes. For dehydration of the gel plugs, shaking in 50 µL of 100% ACN for 15 minutes was performed followed by drying the gel plugs in speed vacuum for 30 minutes at 4 °C. For enzymatic digestion, the gel plugs were incubated with 25 µL of 6 ng/ µL trypsin in 50 mM ammonium bicarbonate at 37 °C overnight.

#### 2.4.2. Extraction

After overnight digestion, the digested products were spun down by vortexing and transferred liquid to the fresh tubes. Adding 50 µL 50% ACN to the tubes, shaking was continued for 15 minutes. Then, the gel plugs were incubated with 50 µL of 100% ACN and shook for another 15 minutes. Transferring the liquid to the previous tubes, the digested samples were completely dried using the speed vacuum at 1000 rpm. The dried tubes were stored at −80 °C and later on de-salting and zip tipping performed.

### 2.5. LC-Chip-MS/MS Q-TOF quantification

All the MS/MS instruments and software used in the present section of the study were from Agilent (Agilent, Santa Clara, CA, USA). Eluted sample obtained from zip tip procedure was dried and 10µL of the lyophilized samples was reconstituted in the first LC mobile phase (0.1% formic acid) in triplicate. The peptides with a Nano-LC 1260 linked directly with an Accurate Mass Q-TOF 6550 containing a Chip-Cube interface Nano-ESI ion source. Polaris High Performance Chip was utilized and enriched the peptides using 360 nl enrichment column followed by their separation using the separation column (C18 reverse phase, 150mm × 75Âµm, 5 µm) with solvent A (0.2% formic acid in water) and a 5–80% gradient of solvent B (0.1% formic acid in acetonitrile) for 34 min with a flow rate of 0.35 µL/min. Mass data acquisition was undertaken at 8 spectra/second in the range of 100–200 m/z and subsequent collision induced dissociation (CID) of the twenty most intense ions. Setting the mass-tolerance of precursor and product ions at 20, MS/MS data acquisition was performed in the range of 200–3000m/z. In order to identify the proteins, the acquired MS/MS data were compared against the UniProtKB/Swiss Prot rat *(Rattus norvegicus)* database using the Spectrum Mill and X! Tandem. The differentially expressed proteins in the different groups were identified using their canonical sequence and proteins having fold change of at least 1.5 times were considered as the deregulated proteins. For validation of the identified proteins, the data were exported to the Scaffold database (version 4.5.1, Portland, USA). Proteins were grouped together if they would share at least two peptides and maintained their threshold level at 95.0% and < 1% false discovery rate (FDR) by the Peptide Prophet *al*gorithm with Scaffold delta - mass correction for the matched peptide-spectra. Proteins that contained similar peptides and could not be differentiated based on MS/MS analysis alone were grouped to satisfy the principles of parsimony. Proteins sharing significant peptide evidence were grouped into clusters. Proteins were annotated with GO terms from NCBI.

### 2.6. Statistical analysis

In the present experiment, label-free relative quantification was performed depending on the regulation of the peptides. For statistical analyses, the data were exported to the Mass Profiler Professional (MPP) software that analyzed depending on the MPP entities, the intensity of the total spectra of the proteins. Setting the baseline of the spectra to the median of the samples, frequencies of the entities were filtered minimally at all the replicates of each treatment. To overcome the complications of false discovery associated with multiple test analyses, ANOVA (P < 0.05) was performed.

## 3. Results and discussion

### 3.1. Eight-armed radial maze study

**Figure 2.**
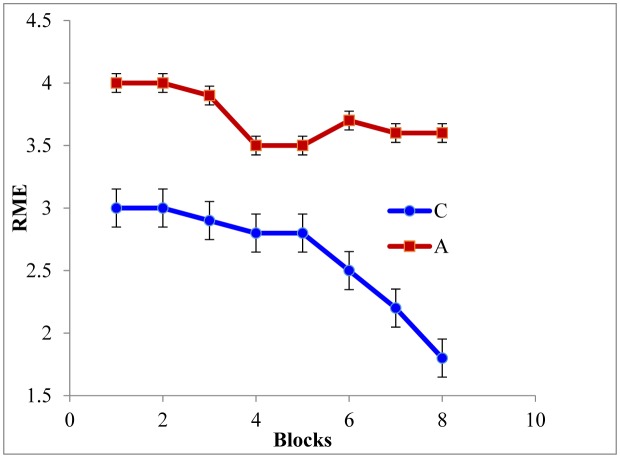
Reference memory error (RME) of the rats. Data of every 6 trials have been averaged over a block and expressed as mean ± SE (n = 3). Data were analyzed with one-way ANOVA and post-hoc Tukey's HSD test (P ≤ 0.05). Here, C = control and A = AD model rats, respectively.

**Figure 3.**
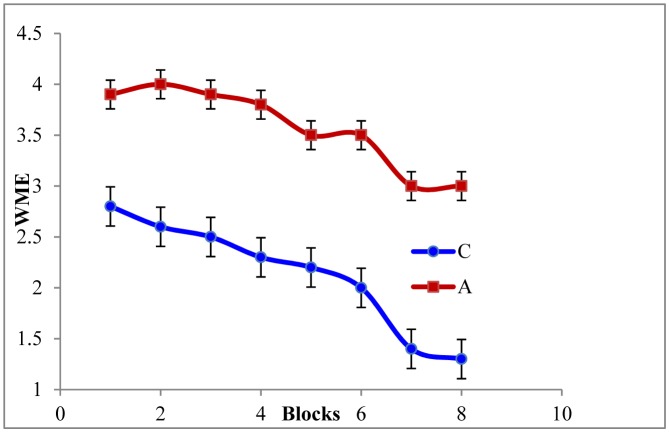
Working memory error (WME) of the rats. Data of every 6 trials have been averaged over a block and expressed as mean ± SE (n = 3). Data were analyzed with one-way ANOVA and post-hoc Tukey's HSD test (P ≤ 0.05). Here, C = control and A = AD model rats, respectively.

**Figure 4.**
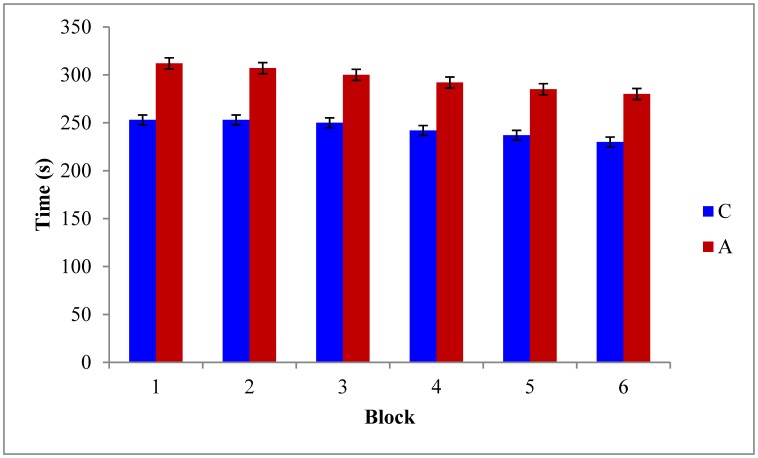
Time spent in exploring the baited arms. Data of every 6 trials have been averaged over a block and expressed as mean ± SE (n = 3). Data were analyzed with one-way ANOVA and post-hoc Tukey's HSD test (P ≤ 0.05). Here, C = control and A = AD model rats, respectively.

As depicted in Figure 2 and Figure 3, the RME [F_(5,42)_ = 11.31, P ≤ 0.05] and WME [F_(5,42)_ = 16.78, P ≤ 0.05] of the AD rats were higher than those of the controls. Tukey post-hoc multiple comparison tests revealed that this discrepancy was statistically significant (P ≤ 0.05). Also the maze latency (time required to explore the baited arms) was higher in the AD rats (Figure 4). These findings represent the declined associative memory of the AD rats. Perhaps, the AD rats could not develop their cognitive maps better and thus failed to extrapolate the location much precisely and swifter than those of the controls. Aβ_1-42_ driven neuro-degeneration in the CA1 region of the hippocampus might have impaired memory in the AD rats [19],[20].

### 3.2. Quantitative analysis of the identified proteins

**Figure 5.**
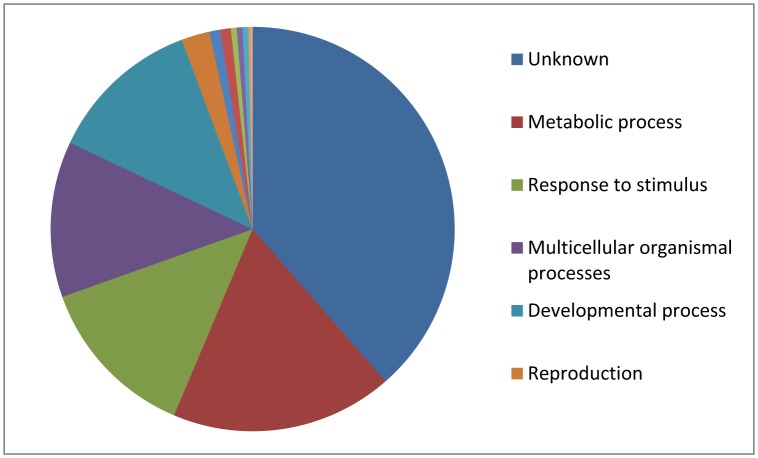
Gene ontology (GO) analysis featuring biological functions of the identified proteins.

Total 822 proteins with protein threshold at 95.0%, minimum peptide of 2 and peptide threshold at 0.1% FDR were identified in the present study. Number of commonly expressed proteins was 361 (Figure 5). Among all the identified proteins (822), 329 were differentially expressed with statistical significance (P < 0.05) (Supplementary Table 1). Among the significantly regulated (P < 0.05) 329 proteins, 289 met the criteria of fold change (LogFC of 1.5) cut off value. Number of proteins linked with AD, OS and hypercholesterolemia was 59, 20 and 12, respectively (Supplementary Table 1). The highest amount of proteins differentially expressed in the AD rats were those involved in metabolic processes (26% increase in the rats) followed by those involved in anti-oxidant activities (Figure 5).

### 3.3. Functional classification of the significantly regulated proteins

#### 3.3.1. Proteins involved in metabolism

Enzymes associated with glucose metabolism (glycolysis, gluconeogenesis and citric acid cycle) were among the most abundantly up-regulated proteins in the present study. Glucose is the main source of energy for the brain cells and alteration in energy production has been linked with AD pathogenesis [21] (Parihar & Brewer, 2007). Present findings of the increased glycolytic enzymes (Hexokinase-1, Glucose-6-phosphate isomerase, Glyceraldehyde-3-phosphate dehydrogenase (GAPDH), L-lactate dehydrogenase B chain), pyruvate metabolizing enzymes (Pyruvate dehydrogenase E1 component subunit alpha and beta), lactate metabolizing enzyme (L-lactate dehydrogenase B chain) are indicative of decreased utilization of glucose for energy production in the AD brains. Glycolytic enzyme GAPDH has other activities such as OS sensor of apoptosis and AD [22]. Minjarez et al (2013) reported differential expression of GAPDH, ubiquitin carboxyl-terminal hydrolase isozyme L1 (UCHL-1) and transferrin as the AD biomarker [23]. UCHL-1 is linked with OS and transferrin with iron regulatory processes. Findings of the present study are in agreement with those of Musunuri et al., (2014) [24].

Altered expression of the enzymes involved in glucose metabolism, might have affected energy generation, ionic balance, membrane asymmetry and transporters in the current experimental subjects. Consequently, impaired neuronal connectivity, transportation, signal transduction and neurotransmission might have resulted in declined cognitive and memory functions of the AD rats [25]. Beyond energy metabolism, decreased rate of glycolysis in the AD subjects has been linked with reduced generation of acetylcholine that impairs memory and learning abilities (Poon et al., 2004) [26]. Among the down-regulated enzymatic proteins, phosphoglycerate mutase 1 and acyl coenzyme A thioesterase 1 and 2 were most notable. Similar observation has been reported by some other researchers [27].

#### 3.3.2. Proteins involved in cellular stress responses

In the AD rat groups, stress proteins of both enzymatic and non-enzymatic types were significantly regulated and differentially expressed. The proteins were of overlapping functionalities against oxidative, nutritional and environmental stressors. Heat shock proteins (HSPs) of variable molecular weight and functions were among the mostly regulated ones. Altered expression of different HSPs in the present study demonstrate their altered levels in the AD pathogenesis and corresponding amelioration in the respective subjects. As AD pathogenesis involves protein misfolding, increased chaperones are expressed in AD brains in response to Aβ and tau proteins [28]. Increased expression of heat shock 70kDa protein 12A (Predicted), isoform CRA_a; heat shock cognate 71 kDa protein, HSP 90 – α, HSP 90 – β, and mitochondrial HSP 60 kDa were noted in the present study that are indicative of different mode and extent of the experimental subjects in response to different stressors. Current findings are in agreement with those of Yao et al. (2007) [29].

#### 3.3.3. Proteins involved in cholesterol metabolism and transportation

a. Apolipoproteins

In rats, apolipoprotein A-IV is the major constituent of HDL and chylomicrons and involved in various functions including cholesterol efflux, stimulation of lipoprotein lipase and cholesteryl ester transfer protein as well as anti-oxidative and anti-inflammatory effects. Apolipoprotein A-IV was downregulated in the AD groups in the present study. Obtained findings are in line with those of Cui et al., (2011) who showed that genetic extirpation of apolipoprotein A-IV aggrandizes Aβ plaque deposition, aggravates neuronal loss and impairs spatial memory in the AD mouse models [30]. Normally, apolipoprotein A-IV functions as the Aβ clearing agent and *in vitro* studies showed that they co-localize with Aβ plaque in the brain [30]. Down-regulation or loss of function of this lipo-protein impairs the Aβ clearing process that ultimately leads towards Aβ deposition [30].

Redox proteomics has identified apo A-I as one of the most promising biomarker of neurodegenearation [31]. Down-regulated expression of apolipoprotein A-I (apo A-I) was observed in the hippocampus of the AD rats. Current findings are compatible with those of Liu et al., (2006) who demonstrated that AD patients' hippocampi and serum possess lowered apo A-I compared with their age matched controls [32]. In the present study, up-regulated expression of apolipoprotein E (apo E) was noticed in the AD rat groups. Apolipoprotein E is the principal carrier of cholesterol and aids in lipid transportation and injury repairment in the brain [32]. Individuals with its ε4 allele are at increased (about 8 fold) risk of AD pathogenesis than those with the ε3 allele and ε2 allele possessing people are at rather protection against AD generation [33]. Though, total apolipoprotein E rather than its any specific allele has been detected in the present study, observed findings are compatible with established notion that apolipoprotein E is involved in AD pathogenesis [34]. Like that of binding with the cell surface receptors, apolipoprotein E transfers lipids to the hydrophobic amyloid-β (Aβ) peptide and aids in the formation of Aβ, cerebral amyloid angiopathy, generation of OS, neurotoxicity, neuroinflammation and neurodegeneration in AD [35],[36]. Choi et al., (2015) had shown that brain level of Apo E and Aβ are regulated by the low-density lipoprotein receptor (LDLR) degrading E3 ubiquitin ligase, idol. Idol had been implicated as the principal regulator of LDLR degradation and an inhibitor to the clearance of brain level of both Apo E and Aβ [37]. Its absence in AD transgenic mice increased brain level of LDLR as well as decreased the levels of both Apo E and Aβ as well as improved Aβ-induced neuroinflammation [37]. Genome wide association studies (GWAS) also have linked Apo E with LDLR and CLU (clusterin) *inter alia* as the cholesterol dysregulatory factors of AD pathogenesis [38]. Thus, increased expression of apo-lipoprotein E might have been among the most favored factors of AD pathogenesis in the current experiment.

b. Apolipoprotein J (Clusterin)

Apolipoprotein J (Clusterin) is a glycoprotein normally expressed in different tissue but highly in the brain and with aging and AD pathogenesis. Elevated expression of apolipoprotein J (clusterin) has been observed in the AD model rats. Clusterin gene, CLU, is the third most risk gene linked with late onset AD and it is responsible about 9% of AD risks [39]. Single nucleotide polymorphism of clusterin gene (CLU) heightens AD risk [39]. Clusterin affects AD pathogenesis in various ways such as promoting Aβ aggregation, neuro-inflammation, apoptosis and controlling lipid metabolism and cell cycle regulation and also through epigenetic mechanism [39]. Hong et al., (2013) reported its elevated expression in the hippocampus of the 5XFAD mice [40].

c. ACAT (acyl-coA: cholesterol acyltransferase)

Elevated expression of ACAT (acyl-coA - cholesterol acyltransferase), enzyme involved in cholesterol esterification, points towards altered cholesterol metabolism in the AD rats [34]. Impaired ACAT and cholesterol metabolism have been linked with increased Aβ production and neurotoxicity [34],[41],[42].

d. Fibrinogen

Fibrinogen was overexpressed in the AD rats' brain of the current study. Fibrinogen is the precursor of fibrin, the primary protein involved in blood clotting. Normally, fibrinogen circulates in plasma and cannot cross the blood brain barrier (BBB). But damaged vasculature of the AD subjects allows its accumulation in the extravascular spaces that further intensifies AD pathogenesis [43]. Elevated level of fibrinogen has been linked with AD propensity [43],[44].

e. Transthyretin

Transthyretin (TTR) co-localizes Aβ in the human AD brains and has been suggested to sequester Aβ, prevent Aβ aggregation and fibrillation and thus protective against AD [45]. Downregulation of transthyretin in the AD rats bears proof of increased Aβ plaque formation.

#### 3.3.4. Proteins involved in anti-oxidative defense

Anti-oxidative proteins up-regulated in the mushroom fed rats were mitochondrial superoxide dismutase [Mn], superoxide dismutase [Cu-Zn], glutathione S-transferase pi and mu, glutathione peroxidase 3; peroxiredoxin-1, -2, -5, -6; catalase, isoform 2 of haptoglobin and mitochondrial stress -70 protein. Mitochondrial superoxide dismutase coverts superoxide anion (O_2_^−^) into H_2_O_2_ and thus reduces the deleterious effect of O_2_^−^ [46]. In the AD patients, its overexpression at the lymphocytic mRNA level has been detected [47]. Studies upon AD mouse model has shown that deficiency of this enzyme stimulates Aβ formation, plaque deposition and tau phosphorylation whereas its increased level demolishes Aβ plaque deposition through diminishing the Aβ-42/Aβ-40 [47]. Thus, to thwart the OS and to maintain synaptic plasticity, upregulation of superoxide dismutase [Cu-Zn] (Sod1) and mitochondrial superoxide dismutase [Mn] might have been achieved through feedback mechanism [48]. Glutathione-S-transferases pi and mu (GSTP1 and GSTM1) are involved in glutathione conjugation and phase II detoxification. The stress regulator Nrf2 regulates these enzymes in response to OS and other stressors. Through protein-protein interaction, GSTP1 lowers the activity of the stress kinase JNK and Cdk5 [49]. Catalase decomposes H_2_O_2_ into H_2_O and molecular oxygen and can also inhibit Aβ aggregation [50]. On the other side, Aβ-42 can thwart anti-oxidative and other functions of catalase by forming “catalase- Aβ-42” complex and generate ROS. Thus, inhibitors of the “catalase- Aβ-42” complex have been recommended for diminishing the OS generated by this interaction [51]. Haptoglobin, another anti-oxidant protein downregulated in the current experiment, is either oxidized and/or downregulated in the AD patients [51]. It is involved in reverse cholesterol transportation and its influence on ApoE and Aβ cross-talk has also been reported [52]. In the current experiment, increased levels of peroxiredoxin-1, -2, -5 and -6 in the AD brains were observed that are indicative of increased OS in the AD model rats [53]. Peroxiredoxins are thioredoxin peroxidases capable of eliminating hydroperoxides through thioredoxin/thioredoxin reductase. Their oxidative modification has been linked with AD and other NDs [54].

## 4. Conclusion

As the AD rats have been found to experience impaired memory and learning activities in the eight armed radial maze study and altered expression of their brain proteins involved in memory and learning related activities, differential expression of the proteins might be attributed to the infused Aβ in the respective model animals. Some of the proteins differentially expressed in the AD model rats are related with glucose and cholesterol metabolism and OS management (such as SOD, GPX, peroxiredoxin, glutathione -S-transferase). This inter-relationship reinforces the OS and altered energy metabolism links of AD pathogenesis. Thus, current findings suggests towards management of OS and energy metabolism in addition with AD therapeutics. Admittedly, the regulated proteins, identified in the current experiment but not previously been studied, warrants extensive exploration for much conclusive remarks. In this endeavor, western blot analysis of the mostly regulated proteins for validation of the current findings is the immediate future aspect of the present study.
