# Supplementary material for: Brain proteomics links oxidative stress with metabolic and cellular stress response proteins in behavioural alteration of Alzheimer's disease model rats
Source: AIMS Neurosci. 2019 Nov 15;6(4):299–315. doi: 10.3934/Neuroscience.2019.4.299 (PMC7179348; doi:10.3934/Neuroscience.2019.4.299)
Supplement: Supplementary file 1 [file neurosci-06-04-299-s001.pdf]

AIMS Neuroscience

AIMS Neuroscience, 6(4): 299–315.
DOI: 10.3934/Neuroscience.2019.4.299
Received: 29 August 2019
Accepted: 28 October 2019
Published: 15 November 2019

http://www.aimspress.com/journal/neuroscience

Research article

Brain proteomics links oxidative stress with metabolic and cellular stress response proteins in behavioural alteration of Alzheimer's disease model rats

Mohammad Azizur Rahman[1,2,*], Shahdat Hossain[1], Noorlidah Abdullah[2] and Norhaniza Aminudin[2,3]

[1] Department of Biochemistry and Molecular Biology, Jahangirnagar University, Savar, Dhaka, Bangladesh
[2] Mushroom Research Centre, Institute of Biological Sciences, Faculty of Science, University of Malaya, Kuala Lumpur, Malaysia
[3] University of Malaya Centre for Proteomics Research, Faculty of Medicine, University of Malaya, Kuala Lumpur, Malaysia

* **Correspondence:** E-mail: azizbmb@juniv.edu.

Supplementary Materials

Table 1. List of differentially expressed and significantly regulated proteins (log fold change > 1.5, p < 0.05).

No.	Protein	Swiss-Prot ID	M. wt. (KDa)	p value	AD vs. C	
					Regulation	Log FC
1	Carbamoyl-phosphate synthase [ammonia], mitochondrial	P07756	165.78	2.13E-04	down	-2.73
2	Serum albumin	P02770	70.73	1.61E-08	up	1.87
3	Alpha-1-inhibitor 3	P14046	165.14	3.78E-04	up	1.77
4	Murinoglobulin-1	Q03626	166.69	0.002112	up	1.62
5	Complement C3	M0RBF1	187.86	0.001387	up	1.77

Continued on next page

No.	Protein	Swiss-Prot ID	M. wt. (KDa)	p value	AD vs. C	
					Regulation	Log FC
6	Heat shock cognate 71 kDa protein	P63018	71.10	8.89E-07	up	2.22
7	Heat shock-related 70 kDa protein 2	P14659	69.93	3.03E-06	up	2.20
8	78 kDa glucose-regulated protein	P06761	72.52	1.84E-04	up	1.87
9	Serotransferrin	P12346	78.56	9.83E-08	up	2.60
10	ATP synthase subunit beta, mitochondrial	P10719	56.35	4.42E-04		
11	Protein Dsp	F1LMV6	334.78	1.06E-11	down	-16.47
12	Glutamate dehydrogenase 1, mitochondrial	P10860	61.76	7.85E-11	down	-15.35
13	Serpina3n-like protein	E0A3N4	48.21	7.49E-04	up	2.41
14	Clathrin heavy chain	F1M779	193.32	8.61E-04		
15	Rat apolipoprotein E protein	Q65ZS7	38.38	5.54E-05	up	1.53
16	60 kDa heat shock protein, mitochondrial	P63039	61.13	2.03E-05		
17	Synapsin-1	P09951	74.16	5.39E-07	up	2.80
18	Synapsin-2	Q63537	63.74	7.18E-05	up	2.33
19	Argininosuccinate synthase	P09034	46.78	0.002866		
20	Junction plakoglobin	Q6P0K8	82.54	8.13E-15	down	-16.85
21	Glial fibrillary acidic protein	P47819	50.01	3.78E-04	down	-2.16
22	Keratin, type II cytoskeletal 8	Q10758	54.02	2.68E-04	down	-3.70
23	Protein Kb15	G3V908	57.95	3.34E-06	down	-2.55
24	Keratin, type II cytoskeletal 73	Q6IG03	61.01	0.003322		
25	Vitamin D-binding protein	P04276	55.14	1.56E-05		
26	Keratin, type II cytoskeletal 5	Q6P6Q2	62.00	9.67E-06	down	-2.55
27	Keratin, type II cytoskeletal 6A	Q4FZU2	59.59	1.16E-06	down	-1.81
28	Keratin, type II cytoskeletal 1	Q6IMF3	65.23	1.49E-06	down	-1.79
29	Keratin, type II cytoskeletal 2 epidermal	M3ZCQ4	69.69	4.72E-04	down	-2.38
30	Neurofilament heavy polypeptide	P16884	115.66	0.006005	down	-1.90
31	Heat shock protein HSP 90-alpha	P82995	85.21	2.71E-04	up	1.64
32	Heat shock protein HSP 90-beta	P34058	83.62	1.84E-04	up	1.71
33	Endoplasmin	A0A0A0MY09	93.18	8.06E-10	up	16.38
34	Neurofilament light polypeptide	P19527	61.39	0.004508		
35	Apolipoprotein A-I	P04639	30.12	2.34E-08		
36	Phosphoglycerate kinase 1	P16617	44.94	8.24E-06		
37	Myosin-4	Q29RW1	223.79	0.022195	up	8.68
38	Protein Myh2	F1LRV9	224.31	0.021982	up	8.68
39	Uncharacterized protein	G3V6E1	220.89	0.021238	up	8.68
40	Hemopexin	P20059	52.09	0.00618	up	2.12

Continued on next page

No.	Protein	Swiss-Prot ID	M. wt. (KDa)	p value	AD vs. C	
					Regulation	Log FC
41	Protein Atp6v1a	D4A133	68.61	0.016075	up	1.88
42	LOC367586 protein	Q5M7V3	51.63	7.19E-06	up	2.18
43	Gamma-enolase	P07323	47.54	6.12E-05	up	1.74
44	Cytochrome b-c1 complex subunit 2, mitochondrial	P32551	48.45	1.80E-05		
45	14-3-3 protein zeta/delta	P63102	27.94	2.73E-04	up	2.19
46	14-3-3 protein theta	P68255	28.06	0.0126	up	1.91
47	14-3-3 protein eta	P68511	28.38	0.00353	up	2.03
48	14-3-3 protein gamma	P61983	28.47	0.019343	up	1.69
49	14-3-3 protein beta/alpha	P35213	28.17	0.008179	up	1.86
50	Keratin, type I cytoskeletal 10	Q6IFW6	56.73	2.55E-05	down	-1.79
51	Keratin, type I cytoskeletal 14	Q6IFV1	52.97	0.026066	down	-1.54
52	Keratin, type I cytoskeletal 17	Q6IFU8	48.41	0.015524	down	-1.82
53	Keratin, type I cytoskeletal 42	Q6IFU7	50.55	0.021279	down	-2.25
54	Keratin, type I cytoskeletal 13	Q6IFV4	48.13	0.002573	down	-2.85
55	Hexokinase-1	P05708	103.60	0.010358	up	1.72
56	Cytosolic 10-formyltetrahydrofolate dehydrogenase	P28037	99.79	1.58E-14		
57	Malate dehydrogenase, cytoplasmic	O88989	36.65	6.06E-05		
58	ADH-like protein	Q8K571	40.63	4.68E-04	down	-1.83
59	Aconitate hydratase, mitochondrial	Q9ER34	86.17	0.002837	up	1.89
60	Vesicle-fusing ATPase	Q9QUL6	83.22	3.10E-04	up	3.99
61	Non-erythroid spectrin beta	Q6XD99	274.49	0.008846		
62	Creatine kinase B-type	P07335	43.01	1.90E-04		
63	Isoform 2 of Haptoglobin	P06866-2	43.10	3.70E-04	up	2.51
64	Hemoglobin subunit beta-1	P02091	16.09	2.52E-05		
65	Hemoglobin subunit beta-2	P11517	16.10	2.85E-04		
66	Glyceraldehyde-3-phosphate dehydrogenase	P04797	36.11	3.42E-06		
67	Calcium/calmodulin-dependent protein kinase type II subunit alpha	P11275	54.68	6.49E-05	up	1.69
68	Calcium/calmodulin-dependent protein kinase type II subunit beta	F1LNI8	65.95	7.09E-05	up	1.80
69	Triosephosphate isomerase	P48500	27.36	9.13E-05	up	4.57
70	Hydroxymethylglutaryl-CoA synthase, mitochondrial	P22791	57.37	2.35E-05		
71	3-oxo-5-beta-steroid 4-dehydrogenase	P31210	37.66	1.62E-12		
72	Aldo-keto reductase family 1, member C1 (Dihydrodiol dehydrogenase 1 20-alpha (3-alpha)-hydroxysteroid dehydrogenase)	Q3MHS3	37.52	3.44E-09		

Continued on next page

No.	Protein	Swiss-Prot ID	M. wt. (KDa)	p value	AD vs. C	
					Regulation	Log FC
73	Protein Akr1c19	D3ZEL2	37.40	8.26E-12		
74	3-alpha-hydroxysteroid dehydrogenase	P23457	37.54	2.78E-10		
75	17beta-hydroxysteroid dehydrogenase	Q2MHD9	37.53	3.31E-12		
76	2',3'-cyclic-nucleotide 3'-phosphodiesterase	P13233	47.67	0.003737	up	1.12
77	Syntaxin-1B	P61265	33.47	3.15E-13		
78	Citrate synthase, mitochondrial	Q8VHF5	52.21	1.20E-06	up	2.07
79	Fructose-bisphosphate aldolase C	P09117	39.68	0.003252	up	2.34
80	Dimethylglycine dehydrogenase	Q5RKL4	96.26	8.18E-10		
81	Alpha-2-HS-glycoprotein	P24090	38.78	2.14E-04		
82	4-hydroxyphenylpyruvate dioxygenase	P32755	45.34	3.43E-08		
83	Glutathione S-transferase Yb-3	P08009	25.85	0.011535	up	2.76
84	Glutathione S-transferase Mu 1	G3V983	26.07	0.015353	up	2.53
85	Glutathione S-transferase Mu 5	Q9Z1B2	27.08	0.048973	up	12.15
86	Methylmalonate-semialdehyde dehydrogenase [acylating], mitochondrial	Q02253	58.26	1.29E-10	down	-14.14
87	Stress-70 protein, mitochondrial	P48721	74.14	2.76E-10	up	17.35
88	Phosphoglycerate mutase 1	P25113	28.95	0.002031	up	2.40
89	Protein Slc25a12 (Fragment)	F1LX07	74.68	0.001387	up	2.34
90	Ornithine carbamoyltransferase, mitochondrial	P00481	39.94	0.012683		
91	Creatine kinase, mitochondrial 1, ubiquitous	Q5BJT9	47.36	1.38E-04	up	2.87
92	Protein disulfide-isomerase A3	P11598	57.08	0.014876	down	-4.50
93	Ceruloplasmin	G3V7K3	121.46	6.50E-08	up	16.66
94	Transthyretin	P02767	15.83	6.59E-05		
95	Protein disulfide-isomerase	P04785	57.35	0.015887	down	-8.78
96	Prohibitin-2	Q5XIH7	33.31	0.006005		
97	Synaptotagmin-1	P21707	47.74	1.15E-04		
98	Thiosulfate sulfurtransferase	P24329	33.63	0.017788	down	-4.82
99	Pyruvate dehydrogenase E1 component subunit beta, mitochondrial	P49432	39.32	5.02E-04		
100	ADP-ribosylation factor 1	P84079	20.75	0.031895	up	10.53
101	ADP/ATP translocase 1	Q05962	33.22	0.002509	up	1.56
102	ADP/ATP translocase 2	Q09073	33.13	0.010116	up	1.52
103	NADH dehydrogenase [ubiquinone] 1 alpha subcomplex subunit 9, mitochondrial	Q5BK63	42.67	3.82E-04		
104	Uricase	P09118	35.16	6.58E-09		

Continued on next page

No.	Protein	Swiss-Prot ID	M. wt. (KDa)	p value	AD vs. C	
					Regulation	Log FC
105	Carbonic anhydrase 3	P14141	29.72	1.35E-13		
106	Isoform 2 of Voltage-dependent anion-selective channel protein 3	Q9R1Z0-2	31.33	0.012938		
107	Voltage-dependent anion-selective channel protein 2	P81155	32.37	0.046547		
108	Isocitrate dehydrogenase [NADP], mitochondrial	P56574	51.42	8.24E-08	up	15.68
109	Sulfotransferase 1A1	P17988	34.19	6.02E-11		
110	Pyruvate carboxylase, mitochondrial	P52873	130.52	5.76E-11		
111	Isoform LMW of Kininogen-1	P08934-2	48.97	0.031895	down	-11.08
112	Dihydrolipoyllysine-residue acetyltransferase component of pyruvate dehydrogenase complex, mitochondrial	P08461	67.68	0.002564	up	1.63
113	Carboxylesterase 1E	Q63108	62.00	0.02349	down	-15.15
114	Neurochondrin	O35095	80.41	2.09E-09	up	17.00
115	Regucalcin	Q03336	33.96	7.05E-10		
116	Syntaxin 1A	Q9QXG3	33.38	1.09E-08		
117	Inter-alpha-inhibitor H4 heavy chain	O35802	103.95	0.007122	up	1.57
118	Glucose-6-phosphate isomerase	Q6P6V0	63.00	7.39E-05		
119	Peroxiredoxin-6	O35244	24.88	2.59E-11	up	16.62
120	Protein Serpina4	Q5M8C3	48.16	2.74E-09	down	-15.24
121	Myristoylated alanine-rich C-kinase substrate	F1LMW7	29.95	8.86E-11	up	14.82
122	LOC500183 protein	Q4KM66	26.03	5.19E-04		
123	Neural cell adhesion molecule 1	P13596	95.46	0.036474	down	-5.39
124	CRP	H6X2V9	25.76	4.70E-05		
125	Aa1249	Q7TMA9	117.83	1.11E-04		
126	Uncharacterized protein (Fragment)	F1LPQ6	39.33	5.62E-04	up	3.35
127	Serine/threonine-protein phosphatase 2B catalytic subunit alpha isoform	P63329	59.33	3.14E-05	up	1.93
128	Annexin	Q66HH8	35.83	0.02747	down	-9.43
129	Sarcosine dehydrogenase, mitochondrial	Q64380	102.64	5.51E-12		
130	Fatty acid-binding protein, liver	P02692	14.33	6.71E-07	down	-17.25
131	Hydroxyacyl-coenzyme A dehydrogenase, mitochondrial	Q9WVK7	34.56	9.88E-11		
132	Elongation factor 1-alpha 1	P62630	50.46	0.048974		
133	Heat shock 70kDa protein 12A (Predicted), isoform CRA_a	D3ZC55	75.17	9.72E-08	up	16.07
134	NADH-ubiquinone oxidoreductase 75 kDa subunit, mitochondrial	Q66HF1	80.38	5.57E-10	up	16.66
135	Clusterin	G3V836	52.05	1.64E-16	down	-15.71

Continued on next page

No.	Protein	Swiss-Prot ID	M. wt. (KDa)	p value	AD vs. C	
					Regulation	Log FC
136	Amino acid transporter	Q8K5B5	61.43	1.00E-05	up	2.12
137	Sodium/potassium-transporting ATPase subunit beta-1	P07340	35.60	3.87E-04		
138	Protein Hoga1	D4A2K1	34.81	6.43E-11		
139	Mitochondrial 2-oxoglutarate/malate carrier protein	P97700	34.42	2.19E-07	down	-16.70
140	ATP synthase subunit O, mitochondrial	Q06647	23.45	3.83E-11	up	17.23
141	Proteasome subunit beta type	G3V7Q6	28.73	2.40E-09		
142	60S acidic ribosomal protein P0	P19945	34.39	2.93E-09		
143	Corticosteroid 11-beta-dehydrogenase isozyme 1	P16232	32.11	1.40E-12		
144	Slc25a3 protein	Q6IRH6	40.08	4.06E-04	up	1.98
145	Glutathione S-transferase	B6DYP7	25.72	3.95E-14		
146	ATP synthase subunit gamma	Q6PCU0	33.11	2.93E-09	down	-16.32
147	Protein Tppp	D3ZQL7	23.72	4.52E-09	up	17.35
148	Acetyl-Coenzyme A dehydrogenase, medium chain	G3V796	46.97	3.78E-11	up	16.44
149	Transitional endoplasmic reticulum ATPase	P46462	90.03	7.98E-09	up	15.11
150	Histone H4	P62804	11.37	7.39E-06		
151	Ras-related protein Rab-2A	P05712	23.71	2.27E-09	up	17.04
152	Alpha-1,4 glucan phosphorylase	B2GV03	97.46	0.041384	up	10.01
153	Glycogen phosphorylase, liver form	P09811	97.94	0.041384	up	9.96
154	ATP synthase F(0) complex subunit B1, mitochondrial	P19511	28.98	3.71E-09	up	18.18
155	Kynurenine--oxoglutarate transaminase 3	Q58FK9	51.61	4.02E-09	up	15.50
156	Carboxylesterase 1D	P16303	62.43	8.73E-10		
157	Cofilin-1	P45592	18.76	0.048974	up	5.87
158	Prothrombin	P18292	71.84	1.44E-08	down	-14.11
159	Succinyl-CoA ligase subunit beta	F1LM47	50.65	1.89E-07	up	14.44
160	ATPase, H+ transporting, V1 subunit E isoform 1, isoform CRA_a	G3V7L8	26.20	4.93E-09	down	-15.88
161	Protein LOC100909412	F7FIH7	21.20	0.021638	down	-18.30
162	Peroxisomal trans-2-enoyl-CoA reductase	A0A096MIT3	33.45	1.99E-09		
163	Protein Aldh8a1	D3ZXY4	54.30	6.05E-13		
164	Inter-alpha-trypsin inhibitor heavy chain H3	Q63416	99.44	2.50E-09	up	16.20
165	Delta-1-pyrroline-5-carboxylate dehydrogenase, itochondrial	R9PY10	165.64	4.86E-12		
166	Bucs1 protein	B5DFA3	66.04	6.22E-11		

Continued on next page

No.	Protein	Swiss-Prot ID	M. wt. (KDa)	p value	AD vs. C	
					Regulation	Log FC
167	Prohibitin	P67779	29.88	0.039269	down	-10.25
168	Cytochrome b5	P00173	15.35	2.68E-09	down	-18.20
169	Carbonic anhydrase 2	P27139	29.28	2.93E-09	down	-16.12
170	S-adenosylmethionine synthase isoform type-1	P13444	44.27	7.71E-09		
171	Superoxide dismutase [Cu-Zn]	P07632	16.08	2.68E-09	down	-17.61
172	D-dopachrome decarboxylase	P80254	13.25	2.63E-08	down	-18.54
173	Peroxiredoxin-2	P35704	21.95	0.009422		
174	Ras-related C3 botulinum toxin substrate 1	Q6RUV5	21.85	5.65E-10		
175	4-aminobutyrate aminotransferase, mitochondrial	P50554	57.20	0.031895	up	17.04
176	Transgelin-3	P37805	22.67	3.46E-13		
177	Carbonic anhydrase 1	B0BNN3	28.36	6.12E-09	down	-15.19
178	Polyubiquitin-C	Q63429	91.20	9.52E-06		
179	ATPase, H+ transporting, V1 subunit D, isoform CRA_c	Q6P503	28.31	0.023331	down	-4.75
180	Nicotinamide nucleotide transhydrogenase	Q5BJZ3	114.61	8.31E-10		
181	Peroxiredoxin-1	Q63716	22.34	0.041384	up	10.70
182	Dihydropteridine reductase	P11348	25.78	4.40E-11		
183	Electron transfer flavoprotein subunit beta	Q68FU3	27.92	5.49E-10		
184	3-hydroxyacyl-CoA dehydrogenase type-2	B0BMW2	27.42	9.91E-09		
185	Beta-soluble NSF attachment protein	F8WFM2	33.90	3.83E-08		
186	Isoform 2 of Clathrin coat assembly protein AP180	Q05140-2	91.66	0.003282		
187	Peroxiredoxin-5, mitochondrial	Q9R063	22.52	2.98E-10	down	-15.52
188	RCG55639, isoform CRA_c	Q5DT04	60.46	1.06E-11		
189	Ubiquitin thioesterase OTUB1	B2RYG6	31.50	1.79E-06		
190	Thy-1 membrane glycoprotein	P01830	18.40	5.99E-10	up	18.40
191	Protein LOC100362298 (Fragment)	D3ZM33	17.97	3.85E-08	down	-17.04
192	Ig lambda-2 chain C region	P20767	11.49	3.47E-08	up	1.76
193	Superoxide dismutase [Mn], mitochondrial	P07895	24.90	0.023666	up	17.78
194	Neuromodulin	P07936	23.72	0.042242	down	-4.57
195	Septin-2	Q91Y81	41.76	7.80E-10		
196	Glycine N-methyltransferase	M0RDH0	32.99	0.021703	up	4.37
197	Ras-related protein Rab-5A	M0RC99	23.85	0.047559	up	10.75
198	SAP	H6X338	26.32	9.78E-16	down	-15.08

Continued on next page

No.	Protein	Swiss-Prot ID	M. wt. (KDa)	p value	AD vs. C	
					Regulation	Log FC
199	Histone H2B	D3ZNH4	15.49	1.79E-04		
200	Protein kinase C and casein kinase substrate in neurons protein 1	Q9Z0W5	50.79	2.63E-08	down	-15.20
201	Glycerol-3-phosphate dehydrogenase [NAD(+)], cytoplasmic	O35077	38.14	6.25E-09		
202	Dihydrolipoyl dehydrogenase, mitochondrial	Q6P6R2	54.61	8.61E-07	down	-13.72
203	Methylenetetrahydrofolate dehydrogenase (NADP+ dependent) 1, methenyltetrahydrofolate cyclohydrolase, formyltetrahydrofolate synthetase	Q5EBC3	101.66	9.63E-10		
204	40S ribosomal protein SA	P38983	32.94	1.27E-08	up	14.88
205	Nucleoside diphosphate kinase B	P19804	17.40	1.52E-06	down	-15.99
206	Corticosteroid-binding globulin	P31211	44.84	0.01144	up	3.83
207	Ras-related protein Rap-1A	P62836	21.33	0.04164	up	5.30
208	Glutathione S-transferase pi	B6DYQ7	23.67	0.021118	up	18.00
209	Long-chain-fatty-acid--CoA ligase 1	P18163	79.20	6.02E-09		
210	Alpha-centractin	P85515	42.73	0.002136	up	1.57
211	Neurofascin	R9PY05	138.87	0.024378	down	-9.67
212	Peptidyl-prolyl cis-trans isomerase A	P10111	18.10	1.63E-11		
213	Ras-related protein Ral-A	P63322	23.72	1.62E-12		
214	Pyridoxal phosphate phosphatase	Q8VD52	33.51	1.70E-07	down	-12.59
215	Liver carboxylesterase 4	Q64573	62.71	7.71E-09		
216	Prostaglandin reductase 1	P97584	36.00	2.07E-08		
217	Leucine-rich repeat-containing protein 59	Q5RJR8	35.33	5.20E-07		
218	Protein Stard10	Q5BJN1	33.30	1.61E-11		
219	Isoform 2 of Septin-5	Q9JJM9-2	44.38	1.09E-11		
220	Ras-related protein Rab-7a	P09527	23.79	1.85E-05	up	17.05
221	Glutathione peroxidase 3	P23764	25.50	3.51E-10	down	-14.39
222	60S ribosomal protein L13	P41123	24.37	7.23E-09		
223	Dihydrolipoyllysine-residue succinyltransferase component of 2-oxoglutarate dehydrogenase complex, mitochondrial	Q01205	49.27	0.048973	down	-9.95
224	Igh-6 protein	Q569B3	69.10	6.13E-08	up	17.87
225	Ras-related protein Rab-11B	O35509	24.60	3.01E-11	up	17.25
226	Adaptor protein complex AP-2, alpha 1 subunit (Predicted)	D3ZUY8	108.76	0.015656	up	8.52
227	AP-2 complex subunit alpha-2	Q66HM2	105.08	1.27E-07	up	15.74
228	10 kDa heat shock protein, mitochondrial	P26772	10.90	0.046951	down	-17.23

Continued on next page

No.	Protein	Swiss-Prot ID	M. wt. (KDa)	p value	AD vs. C	
					Regulation	Log FC
229	Excitatory amino acid transporter 1	P24942	59.87	5.51E-10	up	17.81
230	Protein LOC100365958	F7FHR5	36.08	2.52E-12		
231	Transcriptional activator protein Pur-alpha	F1LPS8	33.85	9.89E-06	up	14.55
232	ATP synthase subunit d, mitochondrial	P31399	18.82	4.37E-15		
233	Protein Syngr3	D4ABK1	21.65	5.17E-05	up	2.32
234	Protein Serpinf2	Q68FT8	55.12	0.043573	up	9.14
235	Protein Dsg1	D3ZM39	115.05	0	down	-17.41
236	Formimidoyltransferase-cyclodeaminase	O88618	59.54	1.37E-14		
237	Histone H2A type 4	Q00728	14.28	6.63E-05		
238	Protein LOC100362339	D4A6G6	16.12	1.09E-05	down	-14.51
239	Ras-related protein Rab-18	Q5EB77	23.26	6.58E-09		
240	40S ribosomal protein S2	P27952	31.52	6.39E-10		
241	T-complex protein 1 subunit gamma	Q6P502	61.22	7.20E-10	down	-14.22
242	Isoform 2 of Vacuolar protein sorting-associated protein 29	B2RZ78-2	21.06	1.99E-11		
243	NADH dehydrogenase (Ubiquinone) Fe-S protein 7	Q5RJN0	24.23	7.25E-11		
244	2,4-dienoyl CoA reductase 1, mitochondrial, isoform CRA_a	G3V734	36.42	3.26E-08		
245	Alpha-aminoadipic semialdehyde dehydrogenase	Q64057	59.26	9.72E-10		
246	Pyruvate dehydrogenase E1 component subunit alpha	D4A5G8	44.31	1.23E-08	up	15.82
247	V-Ki-ras2 Kirsten rat sarcoma viral oncogene homolog	A0JN17	21.72	4.28E-10		
248	Vesicle associated membrane protein 2B	Q9WUW2	14.57	2.49E-11	up	17.92
249	Protein RGD1561381	D3ZZQ8	16.93	0.012683	up	18.01
250	Alcohol dehydrogenase [NADP(+)]	P51635	36.73	7.23E-09		
251	Microsomal glutathione S-transferase	B6DYQ4	17.53	1.67E-12		
252	Calmodulin	P62161	16.84	6.58E-06	up	14.19
253	Tenascin R, isoform CRA_b	A0A096MJE6	151.94	1.20E-09	up	17.23
254	Catechol-O-methyltransferase	F2W8B0	29.83	1.57E-10		
255	Protein LOC100911847	Q6PDV6	16.44	2.74E-09	down	-16.89
256	Aldose reductase	P07943	36.25	0.026749	down	-9.56
257	ATPase, H+ transporting, lysosomal 38kDa, V0 subunit d1	Q5M7T6	40.76	5.74E-06	up	1.50
258	Reticulon-3	Q6RJR6	102.09	8.51E-09		
259	RCG62292, isoform CRA_a	B5DEL9	22.13	1.56E-08		

Continued on next page

No.	Protein	Swiss-Prot ID	M. wt. (KDa)	p value	AD vs. C	
					Regulation	Log FC
260	Isocitrate dehydrogenase [NAD] subunit alpha, mitochondrial	Q99NA5	40.07	5.22E-08	up	16.78
261	Histone H3.1	Q6LED0	15.52	2.15E-09	down	-17.58
262	NADH dehydrogenase [ubiquinone] flavoprotein 2, mitochondrial	P19234	27.72	3.01E-11	up	15.95
263	Isoform Syn2 of Alpha-synuclein	P37377-2	15.87	1.05E-11		
264	Isoform Gamma-2 of Serine/threonine-protein phosphatase PP1-gamma catalytic subunit	P63088-2	39.25	4.55E-08		
265	Cytosol aminopeptidase	Q68FS4	56.55	8.73E-11		
266	Synaptogyrin-1	Q62876	25.90	0.038714	down	-10.26
267	RCG25732, isoform CRA_b	B5DEM5	23.89	6.22E-11		
268	NAD-dependent protein deacetylase sirtuin-2	Q5RJQ4	39.95	0.034961	up	5.26
269	Glutathione peroxidase 1	P04041	22.33	1.73E-13		
270	Aflatoxin B1 aldehyde reductase member 2	Q8CG45	41.13	3.46E-13		
271	Park7 protein	Q5BKC3	23.02	2.52E-12		
272	Hyaluronan and proteoglycan link protein 1	A1A5N6	40.89	0.02627	down	-10.20
273	3-hydroxyanthranilate 3,4-dioxygenase	P46953	32.87	5.62E-11		
274	40S ribosomal protein S8	Q6TXJ0	27.00	2.13E-07		
275	Protein disulfide-isomerase A6	Q63081	48.57	3.44E-09		
276	Dodecenoyl-Coenzyme A delta isomerase (3,2 trans-enoyl-Coenzyme A isomerase)	Q68G41	32.38	1.50E-07		
277	Synaptophysin	P07825	33.60	1.22E-12	up	16.92
278	Reticulon-1	Q64548	83.40	9.38E-11	down	0.00
279	Beta-ureidopropionase	Q03248	44.61	3.50E-12	up	17.07
280	4-nitrophenylphosphatase domain and non-neuronal SNAP25-like protein homolog 1 (C. elegans), isoform CRA_b	G3V728	33.63	1.22E-12		
281	Calreticulin	P18418	48.17	4.55E-09		
282	Pgm1 protein (Fragment)	A1A5L2	63.63	1.43E-09	down	-14.45
283	Visinin-like 1	Q56A29	22.34	1.02E-08		
284	ES1 protein homolog, mitochondrial	P56571	28.51	6.39E-11		
285	D-beta-hydroxybutyrate dehydrogenase, mitochondrial	P29147	38.60	2.19E-10		
286	Isovaleryl-CoA dehydrogenase, mitochondrial	P12007	46.89	1.69E-06	up	16.95
287	Protein LOC100364509	D3ZV50	22.76	9.76E-07		

Continued on next page

No.	Protein	Swiss-Prot ID	M. wt. (KDa)	p value	AD vs. C	
					Regulation	Log FC
288	Protein LOC301861	D3ZEF5	44.32	8.61E-04	up	1.56
289	Cytosolic acyl coenzyme A thioester hydrolase	Q64559	43.19	0.037845	down	-9.31

AIMS Press

© 2019 the Author(s), licensee AIMS Press. This is an open access article distributed under the terms of the Creative Commons Attribution License (http://creativecommons.org/licenses/by/4.0)
